# The transcriptional landscape of the giant freshwater prawn: Embryonic development and early sexual differentiation mechanisms

**DOI:** 10.3389/fendo.2022.1059936

**Published:** 2022-12-07

**Authors:** Nufar Grinshpan, Faiza A.A. Abayed, Melody Wahl, Hadas Ner-Gaon, Rivka Manor, Amir Sagi, Tal Shay

**Affiliations:** ^1^ Department of Life Sciences, Ben-Gurion University of the Negev, Beer-Sheva, Israel; ^2^ The National Institute for Biotechnology in the Negev, Ben-Gurion University of the Negev, Beer-Sheva, Israel

**Keywords:** Differential splicing, embryonic development, freshwater prawn, *Macrobrachium rosenbergii*, sexual differentiation, sexual dimorphism, transcriptional changes

## Abstract

The giant freshwater prawn pj*Macrobrachium rosenbergii* is one of the best studied species in aquaculture. However, the transcriptional changes associated with embryonic development and the sexual differentiation mechanism of *M. rosenbergii* remain to be elucidated. To characterize the embryonic development of this prawn and to determine whether differential expression and differential splicing play roles in the early sexual differentiation of *M. rosenbergii*, we profiled five developmental days of male and female embryos by RNA sequencing. We identified modules of co-expressed genes representing waves of transcription that correspond to physiological processes in early embryonic development (such as the maternal-to-zygotic transition) up to preparation for life outside the egg (development of muscles, cuticle etc.). Additionally, we found that hundreds of genes are differentially expressed between sexes, most of them uncharacterized, suggesting that the sex differentiation mechanism of *M. rosenbergii* might contain clade-specific elements. The resulting first-of-a-kind transcriptional map of embryonic development of male and female *M. rosenbergii* will guide future studies to reveal the roles of specific genes and splicing isoforms in the embryonic development and sexual differentiation process of *M. rosenbergii*.

## Summary statement

Insight into the embryonic development of the giant river prawn is provided by following genes whose expression or splicing changes during embryonic development and between sexes.

## Introduction

The giant freshwater prawn, *Macrobrachium rosenbergii* – the subject of this study and an important aquacultural product the world over – is a highly studied crustacean species by virtue of its sexual plasticity. Males have the ZZ genotype, and females the WZ genotype (1). The life cycle of *M. rosenbergii* comprises four phases, embryo, larva, post-larva and adult (2, 3). The embryonic phase lasts approximately 17 days, and the larval phase varies in length and includes 11 developmental stages (4). During the embryonic and larval phases there is no phenotypic way to distinguish between the sexes, the sex of the embryo/larva can be determined only molecularly *via* sex-specific markers (5). During the post-larval phase, secondary sexual characteristics, such as the *appendix masculina* (6) and genital openings, start to develop (7). The adult prawn presents typical secondary sexual characteristics, and the morphological differences between males and females are clearly visible. Although phenotypic sexual dimorphism is evident only in the post-larval stages, it is likely that the molecular initiation of the sexual differentiation process starts in the earlier embryonic stages (8). However, despite considerable knowledge about the prawn’s phenotypic embryogenesis and sexual differentiation, transcriptional differences during embryogenesis and sexual differentiation have not yet been studied, and their molecular mechanisms are unknown.

One of the techniques best suited to unravelling embryogenesis and early sexual differentiation mechanisms is differential gene expression analysis. This technique may be used to reveal differences between physiological conditions, morphologies (9), tissues, developmental phases, or sexes, but its application in Crustacea is yet to be fully exploited. In those studies that have been conducted in Crustacea, differential gene expression analysis has, for example, been used to link gene expression to the timeline of biological changes during the molt cycle of the adult crab *Portunus pelagicus* (10), but to date embryonic development in this species has been studied only morphologically and biochemically (11, 12). In other studies, differential gene expression between developmental stages and during sexual maturation has been investigated in the orange mud crab *Scylla olivacea* (13), and in *M. rosenbergii* gene expression has been studied in relation to the immune system and to response to stress (14, 15). However, differential gene expression during embryonic development in *M. rosenbergii* is yet to be characterized at the transcriptomic level.

Alongside differential gene expression, differential splicing can also lead to functional changes at the phenotypic level. For example, in the fruit fly *Drosophila*, there do not appear to be any changes in splicing patterns between different time points across development, but differential splicing between different tissues has been shown in several genes (16–19). Among the studies that have been conducted in Crustacea, one – which profiled only one embryonic stage in the Pacific white shrimp *Penaeus vannamei* (also known as *Litopenaeus vannamei*) – showed that alternative splicing occurred in more than 30% of the coding genes, but splicing changes during embryonic development were not studied (20). Thus, the role of splicing in embryonic development of crustaceans also remains unknown.

The mechanisms of sexual determination and sexual differentiation, which have been studied extensively across the animal and plant kingdoms, are known to vary considerably between species (21, 22). In most crustaceans, sex is determined by the ZZ/WZ sex heritability system [like some amphibians, reptiles, and birds (23, 24)]. Crustacean species – like other arthropods – may be gonochoristic, hermaphroditic (25, 26), or even parthenogenetic (27, 28), with some species also having intersex individuals (7, 29). Unlike some crustacean species that have the ability to change sex at maturity (30), *M. rosenbergii is* a gonochoristic species, with its sex being determined by the chromosomal composition after fertilization (31). Nonetheless, the phenotypic sex of *M. rosenbergii* can be converted from male to female and vice versa (32, 33), with both types of conversion protocol resulting in fully reproductive adults. Sex conversion while conserving chromosomal status allows the creation of same-sex populations of *M. rosenbergii* (32, 34, 35) – and also of other crustacean species – with same-sex populations offering numerous aquacultural and ecological advantages, including homogeneous culture size (36), maximized yield at harvest (37) and the environmental safety conferred by using monosex populations as biological control agents that do not impose a threat of becoming invasive species (38). Importantly, for basic research, same-sex populations can be leveraged for the study of sexual determination at early developmental stages, during which it is impossible to sort embryos according to sex. However, currently available sex-conversion protocols for *M. rosenbergii* have two inherent drawbacks—they can be performed only during a specific short time window, namely, at the beginning of the post-larval phase, and they require manual labor. Methods are therefore required for the high-throughput creation of same sex populations, but their development is hampered by lack of knowledge about early molecular mechanisms of the sexual differentiation of the prawn.

Differences in gene expression levels between sexes have been examined in numerous organisms, including plants (39), mammals (40) and particularly arthropods (41). In some arthropods, sexual differentiation is regulated not only by differentially expressed genes, but also by differentially spliced genes (42, 43). For example, it was shown in *Drosophila* that genes regulating sex determination are differentially expressed between males and females (44) and that sex differentiation and sexual speciation are regulated by alternative transcripts (45). In those studies in *Drosophila*, it was shown that sex-specific isoforms of the *doublesex* gene (*dsx*) regulate sexual differentiation (42) and that other genes taking part in the sexual differentiation mechanism are also under sex-specific splicing regulation, including *sex-lethal (sxl)*, *transformer (tra), transformer-2 (tra2)* and *intersex* (*ix*) genes (43). Studies in the crustacean species *Cherax quadricarinatus*, the red claw crayfish, identified three splicing products of *Tra2* that affect sexual differentiation (46). In *M. rosenbergii*, evidence of alternative splicing was drawn from investigations of two isoforms of the hyperglycemic hormone (47), which is regarded to be one of the upstream regulators of the insulin-like androgenic gland (IAG) hormone that is involved in sexual differentiation (6). However, silencing of the hyperglycemic hormone had no effect on IAG levels (48), and therefore it is not clear whether the IAG hormone is affected by the hyperglycemic hormone or its alternative splicing isoforms.

As indicated above, the extent of changes in gene expression and splicing between male and female *M. rosenbergii* are yet to be determined. In the present study, we identified and characterized changes in gene expression levels and splicing patterns both during embryonic development and between sexes of *M. rosenbergii* with the aim to gain better understanding of embryonic development and the early sexual differentiation mechanisms of this prawn.

## Results

### Gene expression along *M. rosenbergii* development

**Figure 1:**
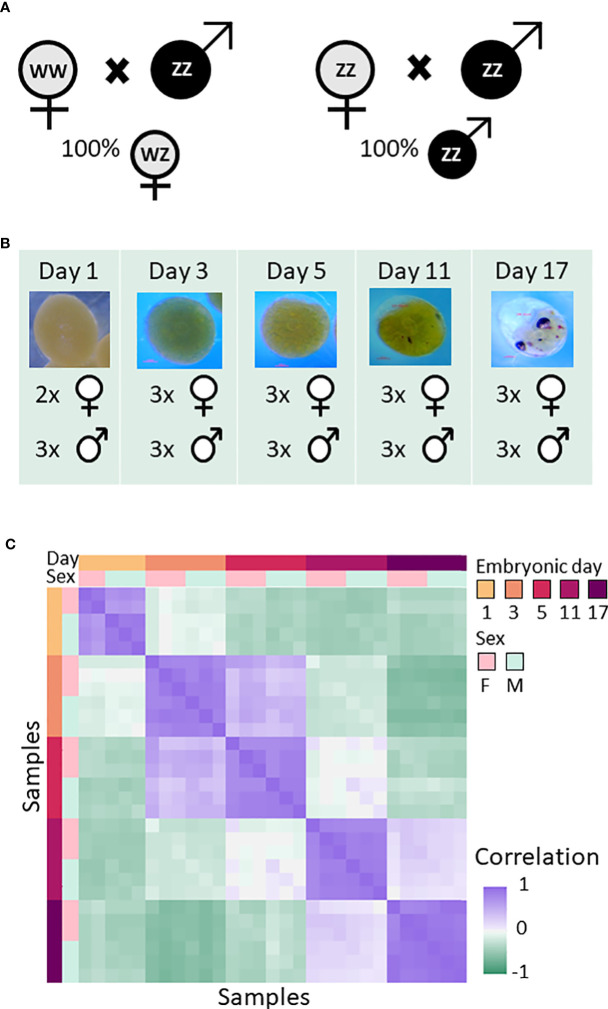
Overall experimental design and resemblance between RNA-seq samples of *M. rosenbergii* embryos sampled on days 1, 3, 5, 11, and 17. **(A)** Creation of same sex progeny. All-female progeny was obtained by crossbreeding a WW female with a ZZ male (left). All-male progeny was obtained by crossbreeding a ZZ neo-female with a ZZ male (right). **(B)** Embryos from different egg-bearing females were collected on days 1, 3, 5, 11, and 17 during embryonic development and pooled. On the figure, the number of pools from each same-sex progeny on each of the five sampling days is indicated next to the sex symbol. **(C)** Correlation matrix between the samples, based on the 2,000 most variable genes across all samples. Samples were sorted by embryonic day and sex. Colors represent Pearson’s correlation coefficient value; see bar on the right of the figure.

To characterize gene expression changes in the two sexes and during embryonic development in *M. rosenbergii*, RNA sequencing (RNA-seq) was used for transcriptional profiling of embryos of both sexes sampled on embryonic days 1, 3, 5 (following the development of the trunk), 11 (showing oval eyes), and 17 (showing rounded eyes) (**Figures 1A, B**). Based on the expression patterns of the 2,000 most variable genes, the transcriptional profiles for the different embryonic days were separated from one another (**Figure 1C**). As expected, the transcriptional profiles for each embryonic day sampled were more similar to one another than to samples from other days, and profiles from each embryonic day sampled were similar to the next day sampled, with the exception of embryonic days 5 and 11, probably due to the longer gap between those two sampling times. Sex did not affect the overall transcriptional profile, with the exception of day 1. Principal component analysis (PCA) also showed that the resemblance in gene expression patterns was higher between samples from the same embryonic day compared to samples from different embryonic days, and that the difference between the sexes was mostly undetectable: Only in samples from day 1 and day 11 embryos was there a detectable difference in gene expression patterns between males and females (**Supplemental Figure S1**). As embryonic development progressed, average expression levels dropped (**Supplemental Figure S2A**), but the number of genes expressed increased (**Supplemental Figure S3**), probably due to the increase in the number of differentiated cell types. Excluding one-day-old embryos, samples of males and females taken on the same embryonic day were similar in terms of the number of genes expressed (**Supplemental Figures S3A, B**) and in the distribution of the expression values (**Supplemental Figure S2B**). Filtering out genes with expression levels lower than the threshold in all samples resulted in the removal of 33432/53119 (62.94%) of the transcripts in the transcriptome (**Supplemental Figure S3B**). The remaining 19687 transcripts (37.06%) were used for all subsequent analyses.

### Gene co-expression patterns reflect embryonic development and sex

**Figure 2:**
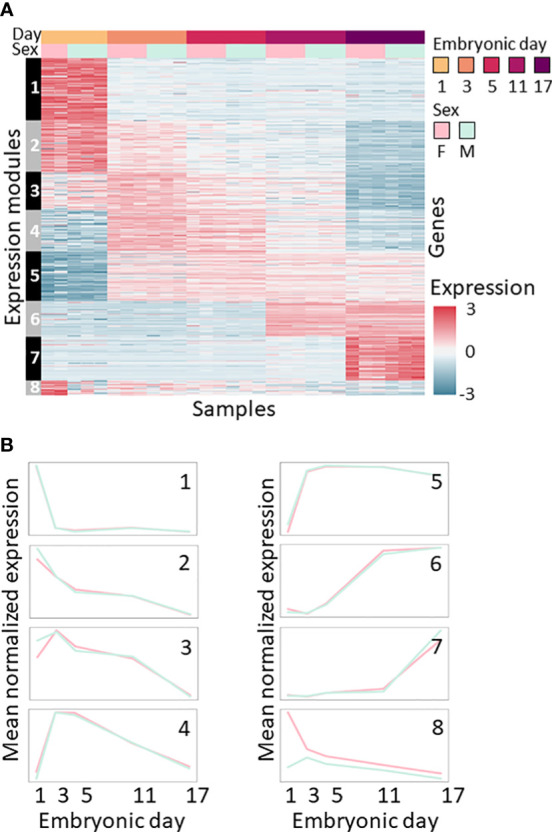
Expression patterns during the embryonic development of *M*. *rosenbergii*. **(A)** Expression level heatmap of the 2,000 most variable genes across all samples. Genes (rows) are sorted according to co-expression modules (marked on left). Samples (columns) are sorted by embryonic day (1–17) and sex, as the bar on the top shows. Colors represent standardized expression levels; see bar on the right. **(B)** Mean expression profiles of each of the eight modules of co-expressed genes identified during embryonic development of *M. rosenbergii*. Profiles are separated by sex, colored as in A, i.e., females in pink, and males in green.

Clustering the 2,000 most variable genes produced eight expression modules of co-expressed genes (**Figure 2**). Modules 1–7 display expression patterns that depend on the embryonic day and are independent of sex. Modules 1 and 2 contain genes that are downregulated with embryonic development. Module 3 contains genes that are upregulated on the third day of embryonic development and then downregulated as embryonic development progresses. Module 4 contains genes that are transiently upregulated during days 3 and 5 of development. Modules 5, 6 and 7 contain genes that are upregulated on days 3, 11 and 17, respectively. Only one module, Module 8, shows differences between males and females, with the genes showing high expression in females and low expression in males, mostly on the first day of embryonic development (**Figure 2**; **Supplemental Tables S1**, **S2**).

The genes that were subjected to clustering were enriched for dozens of biological functions compared to the entire transcriptome (**Supplemental Table S3**), and four expression modules, namely, Modules 2, 3, 4 and 7, were functionally enriched (**Supplemental Table S3**).

### Differentially expressed genes between sexes

**Figure 3:**
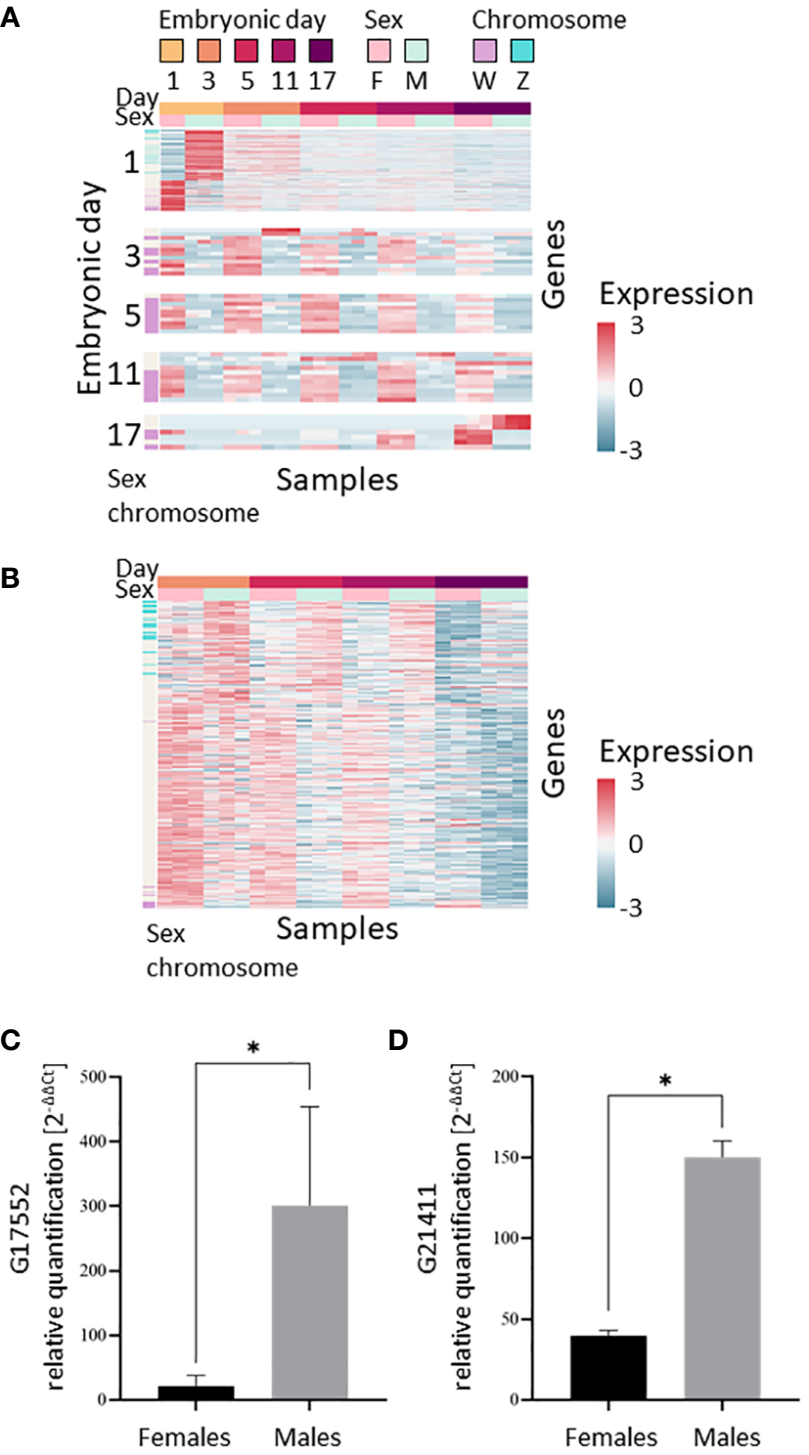
Transcriptional differences between the sexes during embryonic development of *M. rosenbergii*. **(A)** Expression level heatmap of the genes that are differentially expressed between males and females on each sampling day (*t*-test FDR<0.05, |fold change|>1). Genes (rows) were separated by the embryonic day in which they displayed differential expression. On each embryonic day, genes were sorted by the *t*-statistic. Genes located on scaffolds that were identified as part of the sex chromosomes are indicated in mauve (W chromosome) or blue (Z chromosome) on the sex chromosome bar on the left. **(B)** Expression level heatmap of the genes that are differentially expressed between males and females during days 3–17 of embryonic development (paired *t*-test, pairing by days, FDR<0.05, |fold change|>1). Genes were sorted by the *t*-statistic. In both A and B, colors represent standardized expression levels; see bar on the right. **(C, D)** Differentially expressed genes *in vitro* validation. Relative quantification of **(C)** g17552 at day 3 and **(D)** g21411 at day 11 in female and male embryos. Error bars represent the standard error of the means, and asterisks represent the statistically significant differences (*P* < 0.05).

Since only one expression module (Module 8) displayed different expression patterns between males and females in the clustering analysis, we thought it likely that the transcriptional differences between the embryonic days masked the differences between the sexes. We therefore used supervised analysis to specifically search for genes that are differentially expressed between the sexes. To identify transient transcriptional differences between the sexes that may appear only on a single developmental day, we searched for genes that were differentially expressed between sexes on each embryonic day sampled. We identified 329 genes (**Supplemental Tables S4**, **S5**) that were differentially expressed on one or more of the embryonic days sampled [*t*-test false discovery rate (FDR) <0.05, |fold change|≥1, **Figure 3A**], with most of these genes (307 genes) being differentially expressed on embryonic day 1. As embryonic development progressed, there was a decrease in the number of genes differentially expressed between the sexes, i.e., 12, 10, 11, and 7 genes on embryonic days 3, 5, 11 and 17, respectively. Of the 329 genes that were differentially expressed between sexes, expression of 130 genes was higher in females (33 of those genes are known to be on the W chromosome and none on the Z chromosome) and expression of 199 genes was higher in males (24 of those genes are known to be on the Z chromosome and none on the W chromosome). One gene was differentially expressed on all embryonic days sampled, and eight genes were differentially expressed on more than one day, all upregulated in females. The validation study of cases of differentially expressed genes demonstrated similar patterns as found in the *in-silico* analyses results. The differential expressed genes g17552 and g21411 were verified by qPCR and found to be male-biased, as predicted by the *in-silico* differential expression analyses. The relative transcript levels of g17552 and g21411 were significantly higher (*P* < 0.05) in males than females on day 3 and day 11, respectively (**Figures 3C, D**).

The genes that were differentially expressed in one-day-old embryos were enriched for the following gene ontology (GO) annotations: multicellular organism development, DNA binding, protein binding, cytoplasm, heart looping, cytosol and ciliary tip (**Supplemental Table S6**). The numbers of differentially expressed genes on later embryonic days were smaller, with no genes or only one gene with GO terms, and therefore functional enrichment of those genes was not tested.

As day-1 samples displayed the greatest difference between the sexes, we posited that this difference could be due to maternal RNA deposited in the embryo. We thus searched for homologs in *M. rosenbergii* of the *Drosophila* transcription factor *Zelda*, which activates the zygotic genome. Our data revealed that the homologs identified, g25172 and g32461, were both slowly downregulated during embryonic development (**Supplemental Figure S4**), indicating that it is unlikely that the maternal-to-zygotic transition is completed in day-1 embryos. We therefore reviewed together all the data for differentially expressed genes on all later embryonic days sampled in an attempt to pinpoint small differences in expression levels that were consistent for all, or most of, the embryonic days sampled. We identified 301 genes that were differentially expressed between males and females across development up to day 17, but excluding day 1 (paired *t*-test, pairing by embryonic day, FDR<0.05, |fold change|≥1, **Figure 3B**). Of those 301 genes, 100 were upregulated in males (20 are on the Z chromosome and none on the W chromosome), and 201 in females (10 are on the W chromosome and none on the Z chromosome). The genes that passed the paired *t*-test were enriched for the nuclear speck and fatty acid biosynthetic process annotations (**Supplemental Table S6**). Overall, 601 genes differentially expressed between males and females were identified in the analysis, as 29 genes were identified in both per-day and paired *t*-tests (**Supplemental Tables S4, S5**).

### Splicing changes during embryonic development and between the sexes

**Figure 4:**
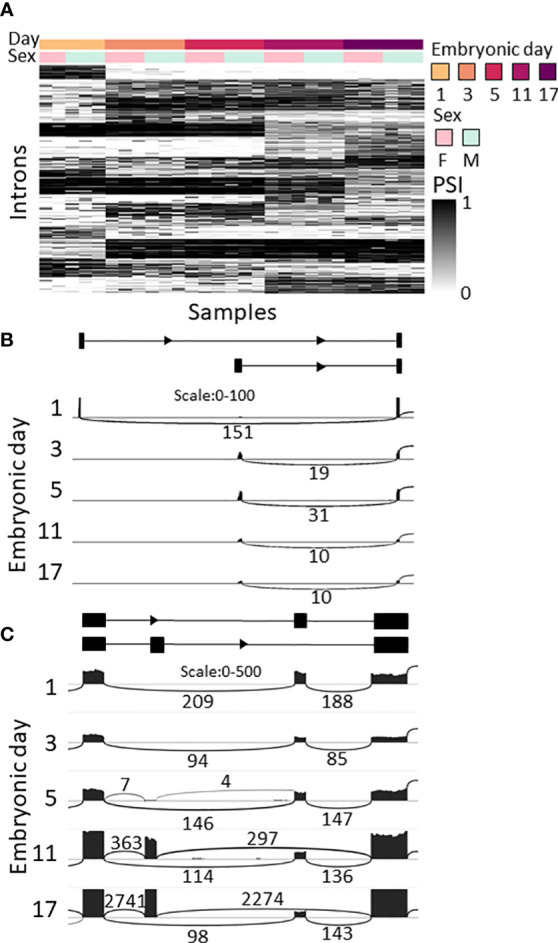
Splicing changes during *M. rosenbergii* embryonic development. **(A)** Splicing ratio, the percent spliced in (PSI), heatmap of the introns that were differentially spliced between embryonic days. Only the 500 introns with the highest Δ are shown. Differentially spliced introns with similar patterns were clustered together. Heatmap colors represent PSI values; see color bar on the right. **(B)** Sashimi plot of alternative first exons in gene g31925, whose ratio changed between day 1 and later embryonic days sampled. Top, diagram of gene structure. Arcs represent splicing junctions, and the numbers on the arcs are the number of junction spanning reads that fall on the junctions. In all subplots, Female_B is displayed as a representative. **(C)** Sashimi plot of mutually exclusive exons in g42168. In samples from 1- to 5-day-old embryos only one transcript is expressed, and in samples of 11- to 17-day-old embryos both transcripts are expressed. Top, diagram of gene structure. Gene structure was inferred from the RNA-seq reads, as this gene is not in the current transcriptome. In all subplots, Female_B is displayed as a representative.

As splicing is known to play a key role in development and sexual determination in *Drosophila* (42, 43), we looked for changes in splicing during embryonic development and between sexes in *M. rosenbergii*. LeafCutter (49) was used for differential splicing analysis due to its lack of dependence on the transcriptome, since it is based on the alignment of the RNA-seq reads to the genome. LeafCutter does not distinguish between splicing events that are the result of alternative promoters, and alternative splicing of transcripts transcribed from the same promoter. Thus, all those events will be considered together and termed differential splicing events (DSE), though the mechanisms behind them are different. We identified 2,054 DSE between embryonic development stages (FDR<0.05) in 948 genes (**Figure 4A**; **Supplemental Table S7**). Some of the changes in splicing during development displayed a binary switch-like pattern, i.e., different transcripts were used on different embryonic days sampled; for example g31925 – an ortholog of a probable acyl-CoA dehydrogenase 6 in *Penaeus vannamei* that is involved in the oxidation of fatty acids in the mitochondria – is transcribed from one promotor on day 1 and from a different, upstream, promotor on days 3–17 (**Figure 4B**). In other DSEs, the ratio between transcripts gradually changed during development. For example, for the mutually exclusive exons on gene g42168, only the downstream exon was expressed on days 1 and 3 (**Figure 4C**). On day 5, the upstream exon was also expressed at a low level, but by days 11 and 17 the upstream exon was expressed at much higher levels than the downstream exon, whose level remained unchanged. The protein predicted to be encoded by g42168 is an ortholog of one of the alpha-actin isoforms in the tick *Ixodes scapularis*. In another gene, g35988, an ortholog of the polychaete gene that regulates morphogenesis in *D. melanogaster* (50), one transcript that skips an exon was expressed throughout development, and expression of another transcript that includes the skipped exon began on day 5. An additional transcript appeared in most of the samples of 17-day-old embryos but was not identified by LeafCutter due to small number of reads (**Supplemental Figure S5A**, transcripts T1, T2 and T3, respectively). PCR of the gene g35988 validated the differential splicing of clu_6480 during development. As expected, on day 3, 5, and 11, two spliced variants were obtained, while on day 1, only one variant was found. On day 17, the g35988 gene comprised three spliced variants (**Supplemental Figure 5B**).

**Figure 5:**
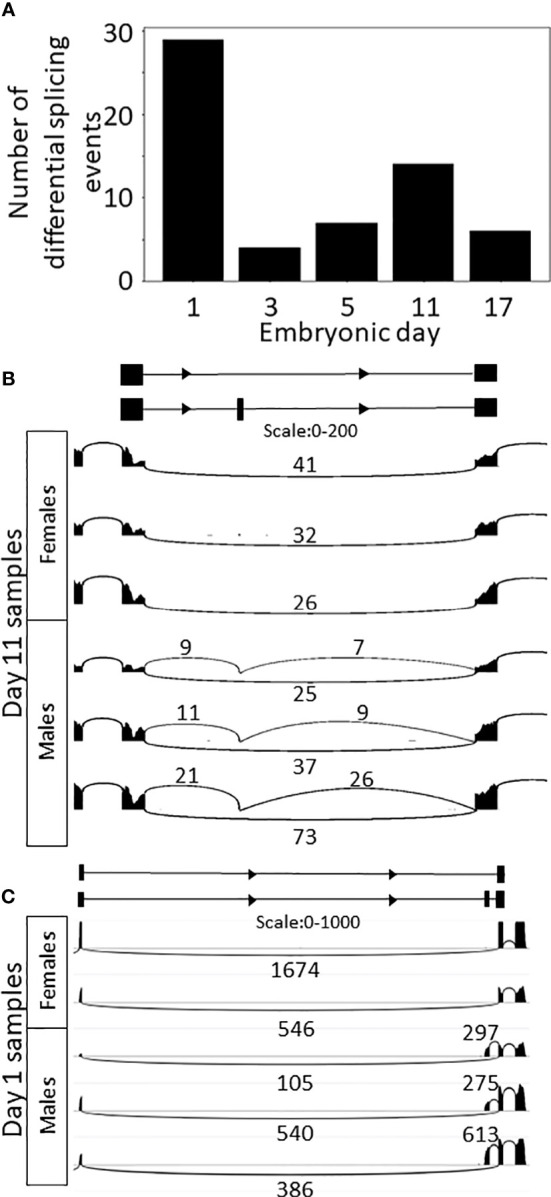
Differential splicing between sexes. **(A)** Bar plot of the number of differential splicing events between sexes on each embryonic day sampled. **(B)** Sashimi plot of skipped exon in gene g44510 in samples of 11-day-old embryos. The inclusive transcript (the bottom one in the diagram) is specific to males. **(C)** Sashimi plot of alternative first exon in g10657 in samples of one-day-old embryos. The bottom transcript in the diagram is specific to males.

Differential splicing analysis between the sexes on each embryonic day identified 90 DSEs (FDR<0.05) in 51 genes (**Figure 5A**; **Supplemental Table S8**). Most of the results were detected on day 1 (48 events), followed by day 11 (20 events) and days 5, 17 and 3 (12, 10, and 4 events, respectively). Four of the events were identified on more than one embryonic day sampled. DSE 11370 showed differential splicing on days 5–17, DSE 9150 on days 1–3, DSE 5988 on days 3–17, and DSE 83 on days 3–5 (**Supplemental Table S8**). Of those 51 differentially spliced genes, only 24 had orthologous genes. DSE 11783 in gene g44510 represents a skipped exon that is differentially spliced between male and female 11-day-old embryos (**Figure 5B**). For this gene, females express only one transcript that skips the exon, but males express two transcripts, one that skips the exon and one that includes the exon. Thus, the transcript that includes the exon is male specific. Gene g44510 is an ortholog of transcription elongation regulator 1 (*TCERG1*), which inhibits the elongation of transcripts from target promotors. Other male-specific transcripts were also detected in the analysis; for example, for g10657, one-day-old female embryos expressed one transcript, whereas male embryos expressed two transcripts, one with the same exons and the other with an alternative upstream first exon (**Figure 5C**). Also detected in the analysis were female almost-specific transcripts, for example, DSE 3387 (**Supplemental Figure S6**), which involves a transcript that does not match any transcript in the transcriptome (LeafCutter is a transcriptome independent method). No ortholog was found for this gene.

## Discussion

Embryonic development in the freshwater prawn *M. rosenbergii* lasts 16–19 days. Although this development has been well characterized phenotypically (51), the transcriptional changes that accompany embryonic development remain unknown. Here, we set out to characterize transcriptional changes during embryonic development, with an emphasis on differences between the sexes, with the aim to identify the genes associated with sexual differentiation. We identified seven modules of co-expressed genes that display development-dependent expression patterns, and one sex-dependent module. In addition, we identified specific genes that display differential splicing both during development and between the sexes. In both the clustering and the differential splicing analyses, most of the changes in the embryonic phase were associated with the embryonic day, and a smaller number were associated with sex. These findings are not surprising, since developmental processes, particularly in the early phases, consume most of the resources of the organism (52). As the sex effect was masked by the developmental processes, supervised analysis was conducted to find differences in expression between the sexes, and, indeed, small groups of genes were found to be differentially expressed between the sexes.

Most of the differences between the sexes were found in one-day-old embryos; however, we had only two samples for one-day-old female embryos, which implies that these results might be less reliable. Nonetheless, many of the genes that were upregulated in males only on day 1 were on the Z chromosome, which has two copies in males and neo-females (ZZ) and one copy in females. Although we cannot rule out the possibility that most of the transcriptional differences between the sexes occur transiently on day 1, i.e., early in embryonic development, it is more likely that day-1 embryos have not yet undergone the maternal-to-zygotic transition, which is conserved in metazoans (53), including crustaceans (54). Thus, day-1 embryos possibly contain a larger quantity of maternal RNA, and thus the differences between the sexes may be attributed to maternal RNA, which is transcribed from neo-females (mature ZZ females) or from mature WW females. Thus, these differences may not reflect transcriptional differences between the embryonic RNA of embryos of different sexes. Prior to the maternal-to-zygotic transition, the embryo genome is not transcribed, and all the RNA is of maternal origin. This time window of the maternal-to-zygotic transition is in accordance with the expression pattern of g25172 and g32461, the homologs of *Zelda*, the activator of the zygotic genome during the maternal-to-zygotic transition in *Drosophila*, and with findings in *Parhyale hawaiensis*, in which only maternal RNA is found in eight-cell embryos (0–9 hours post fertilization), whereas in 32-cell embryos (9.5–12 hours post fertilization) zygotic RNA is already being transcribed (54).

In the splicing analysis between sexes, more DSEs were found on day 11, compared to the other embryonic days sampled, and in the PCA only day-11 samples were separated by sex (excluding one-day-old embryos). There are number of processes in the development of *M. rosenbergii* that are known to occur around the eleventh day of embryonic development, such as the development of the oval eye (days 8–14) and morphological changes in the trunk and caudal portion (51). Thus, it is possible that the processes initiated around day 11 of embryonic development could include early sexual differentiation.

The *M. rosenbergii* genome was sequenced only recently (55), and many of its genes have not yet been annotated. Therefore, we assigned protein orthologs based on the longest reading frame of each transcript, but some of the transcripts of *M. rosenbergii* were not similar to sequences known from other species. Additionally, the genomes and transcriptomes of most species evolutionarily close to *M. rosenbergii* that have been sequenced (56, 57) are also only partially annotated. Another possible reason for the lack of orthologs is that the sexual differentiation mechanism of *M. rosenbergii* is unique and the genes that take part in its cascade may be specific to this species or its clade.

Module 1 includes genes that are upregulated during the first day of embryonic development. For example, g14137 an ortholog of the gene *DIB*. Mutations in *DIB* in *Drosophila* embryos led to problems in larval morphogenesis such as head involution, dorsal closure and gut development (58). The gene g26512 is an ortholog of *STEP*, which is part of the vascular endothelial growth factor pathway that has a crucial role in the eye development in *Drosophila* (59). The genes that were upregulated at the beginning of embryonic development (Module 2, one- and three-day-old embryos) were enriched for several GO terms that were previously reported to be upregulated in early embryonic development, for example, RNA polymerase II cis-regulatory region sequence-specific DNA binding (60), transcription (61), and protein kinase binding that is associated with cell transformation and division (62). One of the genes in this module is an ortholog of the transcription factor PITX2 which has a role in later development of left-right organ asymmetry in vertebrates (63). Gene g42510 is an ortholog of *RDX* in *D. melanogaster*, and is crucial to eye development and chromosomes segregation (64). Genes that were upregulated around the third day of embryonic development (Module 3) were enriched for DNA replication, in accordance with increased number of cells (51, 65, 66). For example, an ortholog of MCM2 which has a role in DNA replication caused by cold stress in *P. vannamei* (67) is a member of module 3. The gene g25945, an ortholog of *Rab11*, is crucial for the development of the *Drosophila* eye (68). Genes that were transiently upregulated during development (Module 4) were enriched for blastocyst development. In *M. rosenbergii*, the blastocyst stage at the beginning of embryonic development (69) is characterized by the formation of the inner cell mass that will subsequently develop into the fetus (70). Two of the genes in module 4 (g38807 and g27228) are orthologues of *JAFRAC1*. Mutation in *JAFRAC1* in *Drosophila* cause germ cells to be left outside of the embryo (71). Module 5 includes genes that are upregulated in days 3-17 of the embryonic development. For example, g27097 is an ortholog of *ALPHA-* SPARC which is part of the trachea development and the anterior Malpighian tubule development in *Drosophila* (72). Module 6 includes genes that are upregulated during days 11-17 of the embryonic development. Some of the genes in this module are related to muscle development, for example the orthologs of *WUPA* and *FLN*. Genes upregulated at the end of the embryonic development (Module 7) were enriched for biological functions that are relevant to that embryonic stage and to the organism’s preparation for the larval phase, without the protection of other mechanisms provided by the egg, such as cuticle formation, muscle development and the detection of UV. When tested for enrichment against all genes, the 2,000 most variable genes that served as the background for module enrichment were also enriched for all the GO terms that were enriched in specific modules, and for additional GO terms, including those related to specific developmental phases, for example, development of the immune system and the nervous system, both of which are known to develop during embryogenesis in other species (73–75).

Although the genes that are differentially expressed between the sexes (Module 8 and *t*-test results, **Supplemental Tables S2, S5**) were assigned orthologs in a higher percentages (**Supplemental Tables S1, S4**) vs. the entire transcriptome, most of their orthologs did not have GO terms associated with them. A possible explanation is that those genes are part of the unique sexual differentiation mechanism of *M. rosenbergii*. As functional enrichment analysis considered only genes that had orthologs with GO terms, no enrichment of sex-related GO terms was detected, even though some of those genes might, indeed, be sex-differentiation related. One gene that is differentially expressed in all sampled time points is g9213, an ortholog gene encoding the aveugle protein. Aveugle is part of the signaling pathway of the epidermal growth factor receptor, which is crucial for the eye development in *Drosophila* (76).

Of the 601 genes that were shown to be differentially expressed between the sexes, 9 were orthologs of zinc finger proteins, which are known to be involved in the development of a specific sex in nematodes and other arthropods (77, 78). One of those genes is g3625 located on the W chromosome, an ortholog of the transcription factor zinc finger E-box binding homeobox protein zag-1, which regulates axon guidance in the neural differentiation in *Caenorhabditis elegance* (79). Few genes that were previously described as DNA repair or immune related in arthropods showed difference in expression between sexes. For example, g17690 which was upregulated in males is an ortholog of the vacuolar protein sorting-associated protein 45 in *P. vannamei*, that was described as part of the immune response genes (80), and was shown in other species from the *Macrobrachium* family as differentially expressed between different molting time phases (81). Two orthologs of the X-ray repair cross-complementing protein 6 (*XRCC6*) which is involved in DNA damage repair pathways and response to radiation in *P. vannamei* (82), were differentially expressed between sexes - g27553 was upregulated in males and g9307 was upregulated in females. Another gene that is upregulated in females is g31721, an ortholog of ATP-citrate synthase, that is part of the immune response of shrimp to the white spot syndrome virus (83). Gene g897 is an ortholog of the lectin protein family member *ERGIC-53*. Lectins participate in the crustacean response to pathogens (84). Those results suggests that there might be differences in immune response between sexes. Another term that was enriched in the genes that were upregulated in males compared to females was the formation of the cuticle. Two of the seven genes that were differentially expressed only in 17-days-old embryos, g34105 and g30699, are orthologs of cuticle proteins CP1158 and one of those seven genes (g31474) is an ortholog of pro resilin. Cuticle proteins and pro resilin are related to the formation of the cuticle and allow for cuticle flexibility (85). Those genes were upregulated in male embryos, suggesting that morphological differences in the cuticle may be detected in a very early stage of the larval phase. Few orthologs of transcription, pre-mRNA processing and splicing factors are also differentially expressed between sexes. For example, orthologs of splicing factors *RBM22* and *PRP1* were upregulated in female embryos and *U2af* was upregulated in male embryos. Those factors might take part in the differential splicing between sexes, but currently it is unknown which genes they regulate in *Macrobrachium* species. In *Drosophila U2af* is part of the splicing mechanism of *tra*, which is one of the sex determination genes (86).

Module 8, which is the only module in which expression patterns were different between the sexes, contained the following types of genes: those whose orthologs regulate translation (g19402 and g2057, orthologs of LSM14); genes that control larval pattern formation (g21244, an ortholog of homeobox); genes that are specific to germ cells of the testis (g16055, ortholog of patched domain, which regulates gene expression in testis); and genes that take part in female gamete development (g2557, the ortholog of the meiosis arrest female protein 1). Some of the genes in Module 8 (9/86) and some of the genes identified by supervised analysis (27/601) have orthologs that are nuclear encoded genes annotated to have a mitochondrion-related function (**Supplemental Tables S2 and S5**). Since it has been suggested that mitochondria play a role in male sex determination in mammals (87) and sexual dimorphism in invertebrates (88), it is thus conceivable that they may also be involved in the sex determination of *M. rosenbergii*.

Splicing analysis was performed to expand our knowledge of the mechanisms active in the embryonic development and sexual differentiation of *M. rosenbergii*. Only a few DSEs between sexes were identified, but those events may include genes involved in the sexual differentiation mechanism. For example, a DSE was identified in g10657, an ortholog of homeobox, which regulates morphogenesis. Out of the 2,145 cases of differential splicing, only 429 (20%) did not map to a gene, and 137 (6.4%) mapped to more than one gene. Visualization of those splicing events showed that the different “genes” are actually a part of a single longer transcript supported by a large number of reads, which indicates that those reads represent real genes and that the current *M. rosenbergii* transcriptome is only partial and can be complemented with additional RNA-seq datasets. At this stage, experimental studies are needed to identify genes that are differentially expressed or spliced between the sexes of *M. rosenbergii* and to understand their influence on the development and sexual differentiation in this prawn.

In conclusion, this study focused on transcriptional changes that mediate embryonic development and presumably the beginning of sexual differentiation in *M. rosenbergii*. Thousands of genes were found to be differentially expressed between the different embryonic days sampled, laying down the foundation for a deeper investigation of embryonic development of crustaceans. *In vitro* study of representative differentially expressed and spliced genes validated the bioinformatics results by demonstrating similar patterns as predicted in the *in-silico* analyses. Although the hundreds of genes that are differentially expressed between the sexes do not provide any clues to the mechanism controlling the sexual plasticity of *M. rosenbergii*, they can be leveraged to guide future functional experimental studies. To the best of our knowledge, this is the first study in a *Macrobrachium* species that provides temporal resolution of the embryonic development and transcriptional sexual dimorphism of *Macrobrachium* embryos.

## Materials and methods

### Embryo transcriptomic library

*M. rosenbergii* females bearing same sex and karyotype embryos (WZ or ZZ) were created as follows. All-female progeny was obtained by crossing ZZ males with WW females, as previously described (32). All-male progeny was obtained by crossing ZZ males with ZZ females, as previously described (33) (**Figure 1A**). Monosex pools of *M. rosenbergii* embryos of each sex were collected in three replicates on five sampling days during embryonic development, i.e., 1, 3, 5, 11 and 17 days after fertilization (**Figure 1B**). *M rosenbergii* embryonic phase lasts 18 days, and in the 18th day the eggs hatch and the larval phase begins. The first and last days, 1 and 17, were chosen to profile the start and end point of the embryonic development, respectively. Assuming that most of the changes take place in the beginning of the embryonic development, right after the sex determination, two time points in the beginning of the embryonic development (days 3 and 5) were sampled, and an additional time point was sampled towards the end (day 11) following the development of the eyes. Total RNA was extracted with the EZ-RNA Total RNA Isolation Kit (Biological Industries) according to the manufacturer’s instructions. Each sample was of 50-70mg of egg mass which was translated to approximately 100-150 eggs. Paired end 100-bp reads were sequenced using Illumina Technology (89). One of the samples of female day 1 was of low quality [%duplicated sequences > 93% and GC content > 55% according to FastQC (90) report, which were high compared to all other samples], and therefore removed from the analysis, resulting in only two replicates for female day 1 embryos.

### RNA sequencing data preprocessing and filtering

RNA-seq reads were mapped to the *M. rosenbergii* genome (55) and the transcriptome assembly based on an embryonic library (89), using HISAT2 (91) with default parameters, thereby generating a BAM file for each sample. In each BAM file, reads were sorted and indexed using samtools (92). Gene expression counts were calculated by RSEM (93) with the flags –paired-end and –star. Counts were transformed to transcripts per million (TPM) (93, 94) using the “bioinfokit.analysis” Python Package (95). TPM values lower than one were replaced by one, and all gene expression values were log2 transformed. An expression threshold of 4 was set by visual inspection of a scatter plot of the replicates (**Supplemental Figure S7**) and the histograms of the abundance of expression values for each sample (**Supplemental Figure S2B**). Genes that were below this threshold in all samples were removed from the analysis.

Pearson correlation coefficients between samples were calculated by Python function corr over the 2,000 genes with the highest standard deviation across all samples. PCA was performed by Matlab function PCA over the same 2,000 genes.

### Clustering analysis

The 2,000 genes with the highest standard deviation across all samples were clustered with the Python KMeans function in the “sklearn.cluster” package with default parameters. The number of expression modules was chosen by elbow heuristics (96). For visualization purposes, each gene expression value was standardized by subtracting the mean for each gene across all samples and dividing by the standard deviation of the same gene across all samples. The average profile of each module in each embryonic day sampled and sex was calculated as the average of the standardized expression values of the genes in the module in all samples of that embryonic day and sex.

### Differential expression analysis

Identification of genes that were differentially expressed between the sexes on each sampling day was performed by a two-sample *t*-test with unequal variances. Only the genes with a minimum expression level of 4 after log2 transformation in at least three (except for two in day 1 analysis) of the samples for a particular sampling day and an expression level range |max-min| ≥ 1 on that sampling day were subjected to the test.

Identification of genes that were differentially expressed between the sexes across embryonic development was done by a paired *t*-test. Since there was a larger difference between males and females in one-day-old embryos compared to all other days in the number of differentially expressed genes (**Figure 3A**; **Table S4**) the paired *t*-test excluded one-day-old embryo samples. Samples were paired by embryonic day. Within each embryonic day, several pairings of samples were tested, with very similar results (data not shown). Only genes with a minimum expression level of 4 after log2 transformation in at least seven of the samples (to ensure that the gene is expressed in at least one sex on more than two days) and an expression level range |max-min| ≥ 1 in all samples tested were subjected to the test. For reporting the results of both tests, the FDR (97) was set to 0.05 and the minimal fold change to 1.

### Differential splicing analysis

Differential splicing analysis was performed to detect alternative splicing events whose frequency was different between embryonic days sampled or between males and females on each sampling day. The analysis was performed using LeafCutter (49), which – being a transcriptome independent method – is capable of identifying differential splicing in genes and exons that are not in the transcriptome. As the *M. rosenbergii* transcriptome is only partially annotated, this method, which is not limited to known genes and splicing events, is more suitable than transcriptome-based methods, such as rMATS (98). LeafCutter defines an alternative splicing event, called a cluster, as a set of two (or more) introns that share a common start or end point. The percent spliced in (PSI,) of an intron is defined as the number of junction spanning reads of that intron divided by the junction spanning reads of all introns in the cluster (**Supplemental Figure S8A**). Differential splicing is indicated by the difference in PSI values between different conditions (ΔPSI, **Supplemental Figure S8B**).

Several filtering criteria for LeafCutter that were effective in reducing false positive findings in practice were applied. For comparisons between embryonic days sampled, the following values for the parameters of LeafCutter were used: M = 10; i = 4; g = 3, i.e., at least 10 junction-spanning reads for at least one of the introns of a cluster in each sample, in at least four samples, and at least three consistent samples in each of the two embryonic days compared (out of five samples for day 1 and six samples for all the other embryonic days sampled). For comparisons between the sexes on each sampling day, the following criteria were used: M = 7; i = 2; g = 2, i.e., at least seven junction-spanning reads for at least one of the introns of a cluster in each sample, in at least two samples, and at least two consistent samples in each sex (out of two for day-1 females, and three for all other conditions). Differential splicing was defined as LeafCutter cluster with FDR<0.05 and a difference in the junction spanning reads fraction (ΔPSI) higher than 0.2 between conditions. Genes of interest were visualized using the Integrative Genomics Viewer (IGV) (99, 100) and Sashimi plots (101) (**Figures 4B, C**, **5B, C**, and **Supplemental Figures S4**, **S5A**).

### Orthology assignment

All sequences were extracted from the *M. rosenbergii* genome according to transcriptomic locations using the RSEM function rsem-prepare-reference (93). For each gene, all open reading frames (ORFs) were detected and translated into amino acids by NCBI’s ORF finder (102). The longest amino acid sequence of each gene was chosen for the subsequent analysis. The blastp option of BLAST (103), which compares protein queries to a protein database, with default parameters was used for all *M. rosenbergii* transcripts as query sequences. Query sequences were compared to the nr protein database in May 2021. Uncharacterized proteins were removed from the results. Overall, there were 33,635,316 orthologs for 49,260 of the 53,119 *M. rosenbergii* transcripts (above 92%). The results originated from 1,802 species in the nr database, but there were very few results for some species. Thus, for assignment to orthologs, we focused on species that are evolutionarily close to *M. rosenbergii* (*Penaeus vannamei* and *M. nipponense*), species that had high number of blast hits (*Tribolium castaneum*, *I. scapularis*, *Branchiostoma floridae*, *Oryzias latipes* and *Nematostella vectensis*), or species whose development and sex differentiation have been extensively studied (*Homo sapiens, Mus musculus* and *D. melanogaster*). Limiting the orthologs to those from these 10 species reduced the number of *M. rosenbergii* transcripts for which orthologs were assigned from 49,260 (92.74%) to 29,543 (55.62%) transcripts. The best hit for every query sequence from each of the above species was extracted for all the genes of *M. rosenbergii.* For each *M. rosenbergii* predicted protein, a gene symbol was assigned using EntrezDirect (104).

### Functional enrichment

There is currently no GO annotation for *M. rosenbergii.* Thus, gene function was predicted by homology. The GO terms associated with each of the orthologs of each gene were extracted from the GO database (105). Orthologs from *O. latipes*, *H. sapiens*, *M. musculus* and *D. melanogaster* were used due to the high number of genes with GO terms. The GO terms were then considered to describe the *M. rosenbergii* gene for the purpose of functional enrichment. GO terms that were assigned more than once to the same *M. rosenbergii* gene (due to being assigned the same term in different organisms) were collapsed to one. Terms that were assigned to only one gene in the tested group were not tested for enrichment. Functional enrichment for each group of genes of interest was calculated by a hypergeometric test using the “stats” package in R (106). Terms that received FDR ≤ 0.05 were considered enriched in the tested gene group. The background for the hypergeometric test for functional enrichment of the modules was the 816 genes that were mapped to unique orthologs having GO terms out of the 2,000 most variable genes serving as the input for the clustering analysis (**Supplemental Tables S1-3**). For the functional enrichment of the differentially expressed genes between males and females, all the genes that underwent the *t*-test were used as the background. Specifically, in the paired *t*-test, the background set included the 2,076 genes with orthologs having GO terms out of the 5,493 genes that passed the thresholds for expression level and minimum range of expression. In the two-sample *t*-test, for each time point, the background was the set of the genes that were assigned orthologs having GO terms out of the genes that passed the filtering and were subjected to the test for days 1, 3, 5, 11 and 17 (1235/5838, 445/2654, 311/2152, 177/1480 and 74/1704, respectively).

### *In vitro* validation

In order to validate the differential expression analysis and the differential splicing analysis, *in vitro* experiments of 3 cases of differentially expressed and spliced genes was performed. RNA was extracted from different embryonic stages (day 1, day 3, day 5, day 11, and day 17) in all-female (32) and all-male (33) progenies as previously described (89). Total RNA was extracted with the EZ-RNA Total RNA Isolation Kit (Biological Industries) according to the manufacturer’s instructions. Complementary DNA (cDNA) was synthesized by a reverse-transcriptase reaction using the qScript cDNA kit (Quanta BioSciences) according to the manufacturer’s instructions containing 1 μg extracted total RNA.

Differentially expressed genes validation of representative genes, g17552 and g21411, was performed by qPCR with specific primers, Universal Probe Library probes (Roche), and the SensiFAST Probe Hi-ROX Mix (BIOLINE). For g17552, 4 cDNA samples from males and 3 cDNA samples from females, both at day 3, were used. For g21411, 6 cDNA samples from males and 6 cDNA samples from females, both at day 11, were used. The relative transcript levels of g17552 were quantified using the forward 5’- TTGTACAATTACTACACCCGTGA-3’ and reverse 5’-AGGCAAGGGTAAGGGCTTT-3’ primers with probe 80 (Roche). g21411 relative quantification was performed using the forward 5’-CCGAAAACCAGTCGATGTCT-3’ and reverse 5’-GTGGCCAGGATTGACACC-3’ primers with probe 144 (Roche). *Mr18S* (GQ131934) was used as a normalizing gene with the specific forward 5’-TAGGTGGTCTCGTGAATGCC-3’ and reverse 5’-TAGGTGGTCTCGTGAATGCC-3’primers with probe 152 (Roche). The qPCR reactions were performed in the QuantStudio Real-Time PCR System, Applied Biosystems (Foster City, CA, United States).

Validation of differential splicing during development was performed by PCR amplification of g35988 (clu_6480). The cDNA samples (2 cDNA samples from each stage) were amplified using gene-specific primers (forward: 5’-TTATCGCCGGGTATAAGTCC-3’and reverse: 5’-ATTCGCAACCCTGAGCATAG-3’) and PCRBIO HS Taq Mix Red according to the manufacturer’s protocol. *M. rosenbergii* β-actin (AF221096) served as a positive control using the following specific primers: Forward: 5-GAGACCTTCAACACCCCAGC-3’ and reverse: 5’-TAGGTGGTCTCGTGAATGCC-3’. PCR products were separated on 1.5% agarose gels.

### Statistical analyses

For the relative quantification by qPCR in the differential expression analysis validation, all data were logarithmically transformed to facilitate proper statistical analysis using Statistica v13.5 software (StatSof Ltd., Tulsa, OK, USA). Normality was tested using the Shapiro-Wilk test, and data of g17552 and g21411 were compared and analyzed using a *t*-test and Mann-Whitney U test, respectively.

## Data availability statement

The data presented in the study are deposited in the NCBI SRA repository, accession number PRJNA906315.

## Author contributions

NG - validation, resources, formal analysis, visualization, software, investigation, and writing - original draft. FA –methodology and data curation. MW - experimental validation. HN-G – software and methodology. RM - resources, writing- review editing, and investigagtion. AS - supervisiong , methodology ,conceptualization, and writing - review editing. TS -supervision, methodology, conceptualization, writing - review, editing, project administration, and funding acquisition. All authors contributed to the article and approved the submitted version.

## Supplementary material

Supplementary Figure 1 Resemblance between samples and conditions. PCA of the samples used in the analysis, based on the 2,000 most variable genes. Days are marked in different colors. Female samples are represented by circles, and male samples, by asterisks.

Supplementary Figure 2 Gene expression distributions. **(A)** The median of the expression levels [log2(TPM)] in each sample, by embryonic day. **(B)** Histograms of gene expression level [log2(TPM)]. Each line is a sample. Samples are colored by embryonic day.

Supplementary Figure 3 Overview of gene expression levels. **(A)** The number of genes that are expressed [log2(TPM)>0] in each sample, by embryonic day. **(B)** The number of genes that are expressed above a chosen expression threshold [log2(TPM)>4] in all samples, by embryonic day. In both A and B, male samples are in green, female samples are in pink.

Supplementary Figure 4 Gene expression pattern of *Zelda* homologs. Log2 transformed expression values of two orthologs of *Zelda*, **(A)** g25172 and **(B)** g32461, across embryonic development, in all samples, by embryonic day. Each dot represents a sample. Male samples are in green, and female samples are in pink.

Supplementary Figure 5 Differential splicing during embryonic development. **(A)** Sashimi plot of skipped exon in gene g35988. In samples from 1-day-old embryos, only one transcript is expressed (T1), in samples of 3- to 11-day-old embryos two transcripts are expressed (T1, T2), and in samples of 17-day-old embryos all three transcripts are expressed (T1, T2, T3). Primers are represented by red arrows. **(B)** *In vitro* validation of the different splice variants of clu_6480 in g35988 (marked in black arrows) during embryonic stages (day 1, 3, 5, 11, and 17; top panel). β-actin served as a positive control (bottom panel).

Supplementary Figure 6 Differential splicing between sexes. Sashimi plot of skipped exon in DSE 3387 in samples of 11-day-old embryos. The inclusive transcript (bottom in the diagram) is specific to females.

Supplementary Figure 7 Quality control of the samples. Scatter plots of expression levels [log2(TPM)] of all genes between samples of embryos of the same embryonic day and same sex. Each dot is a gene. Red line is the x=y diagonal.

Supplementary Figure 8 Leafcutter nomenclature. **(A)** A Leafcutter cluster is composed of all introns that share the same start or end location; here, introns 1-3. For each intron in each sample, Leafcutter assigns a percent spliced in (PSI) value, which is the proportion of the junction spanning reads that fall on this intron out of all junction spanning reads that fall on the introns in this cluster. **(B)** Differential splicing is identified by the difference in PSI between conditions, Δφ.
