# Supplementary figures and images for: The transcriptional landscape of the giant freshwater prawn: Embryonic development and early sexual differentiation mechanisms
Source: Front Endocrinol (Lausanne). 2022 Dec 7;13:1059936. doi: 10.3389/fendo.2022.1059936 (PMC9767951; doi:10.3389/fendo.2022.1059936)

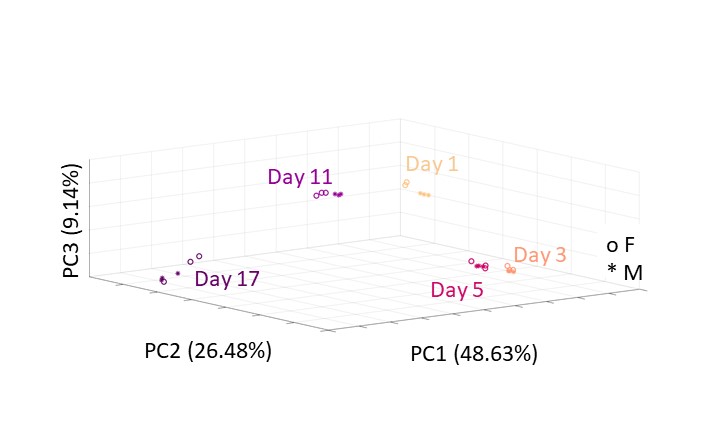

Supplement: Supplementary Figure 1 — Resemblance between samples and conditions. PCA of the samples used in the analysis, based on the 2,000 most variable genes. Days are marked in different colors. Female samples are represented by circles, and male samples, by asterisks. [file Image_1.jpeg]

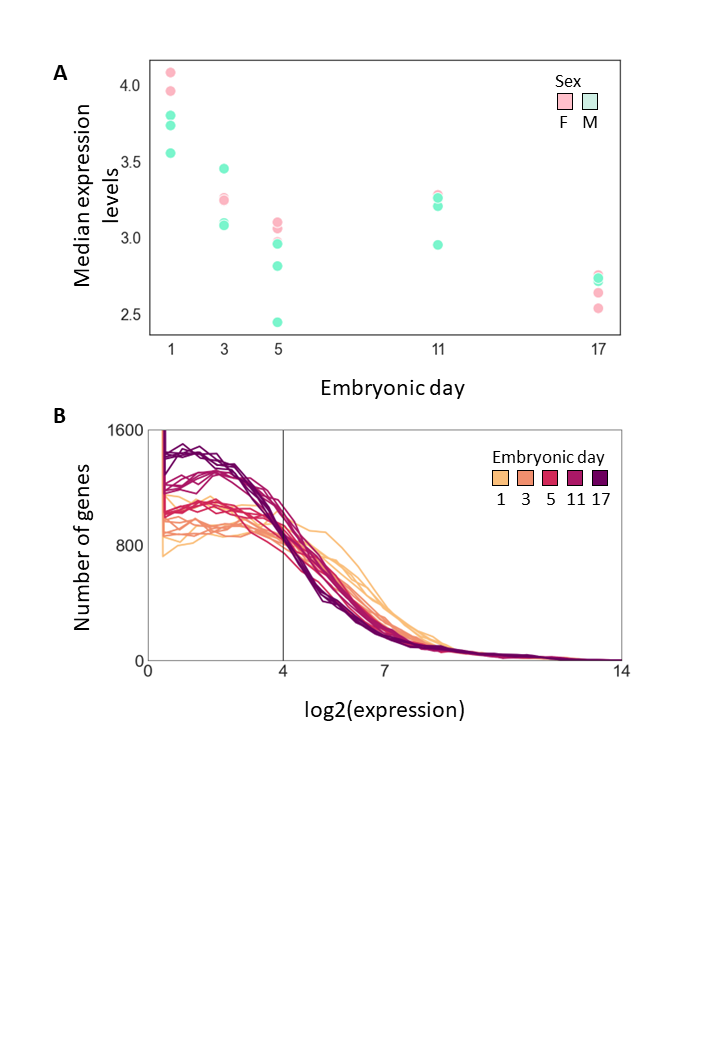

Supplement: Supplementary Figure 2 — Gene expression distributions. (A) The median of the expression levels [log2(TPM)] in each sample, by embryonic day. (B) Histograms of gene expression level [log2(TPM)]. Each line is a sample. Samples are colored by embryonic day. [file Image_2.tif]

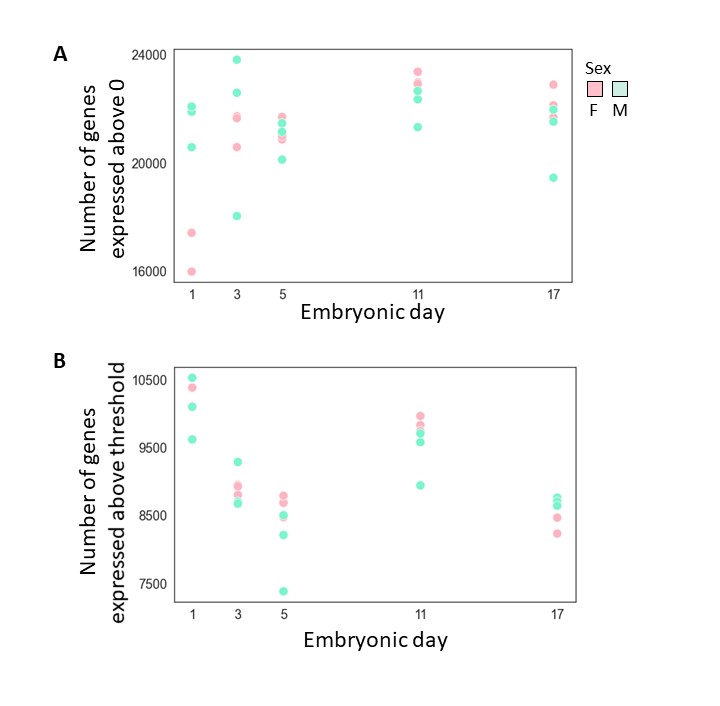

Supplement: Supplementary Figure 3 — Overview of gene expression levels. (A) The number of genes that are expressed [log2(TPM)>0] in each sample, by embryonic day. (B) The number of genes that are expressed above a chosen expression threshold [log2(TPM)>4] in all samples, by embryonic day. In both A and B, male samples are in green, female samples are in pink. [file Image_3.jpeg]

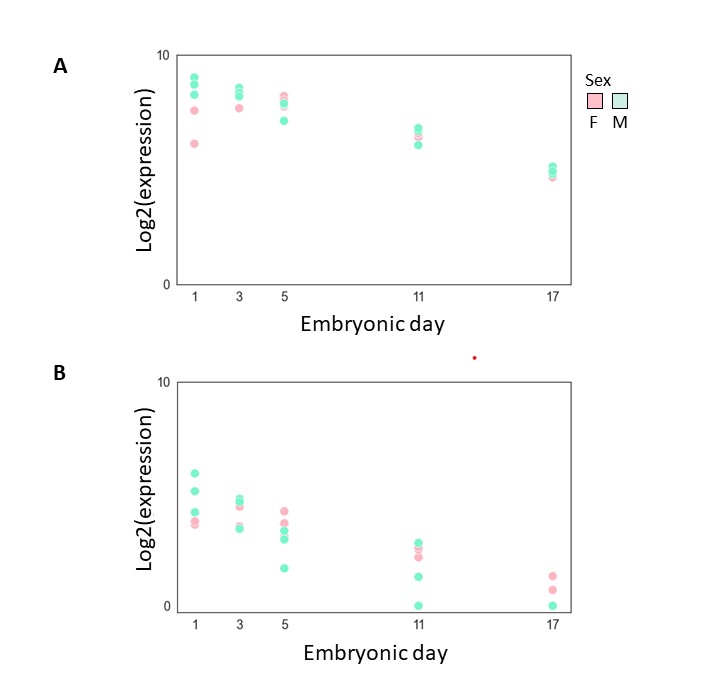

Supplement: Supplementary Figure 4 — Gene expression pattern of Zelda homologs. Log2 transformed expression values of two orthologs of Zelda, (A) g25172 and (B) g32461, across embryonic development, in all samples, by embryonic day. Each dot represents a sample. Male samples are in green, and female samples are in pink. [file Image_4.jpeg]

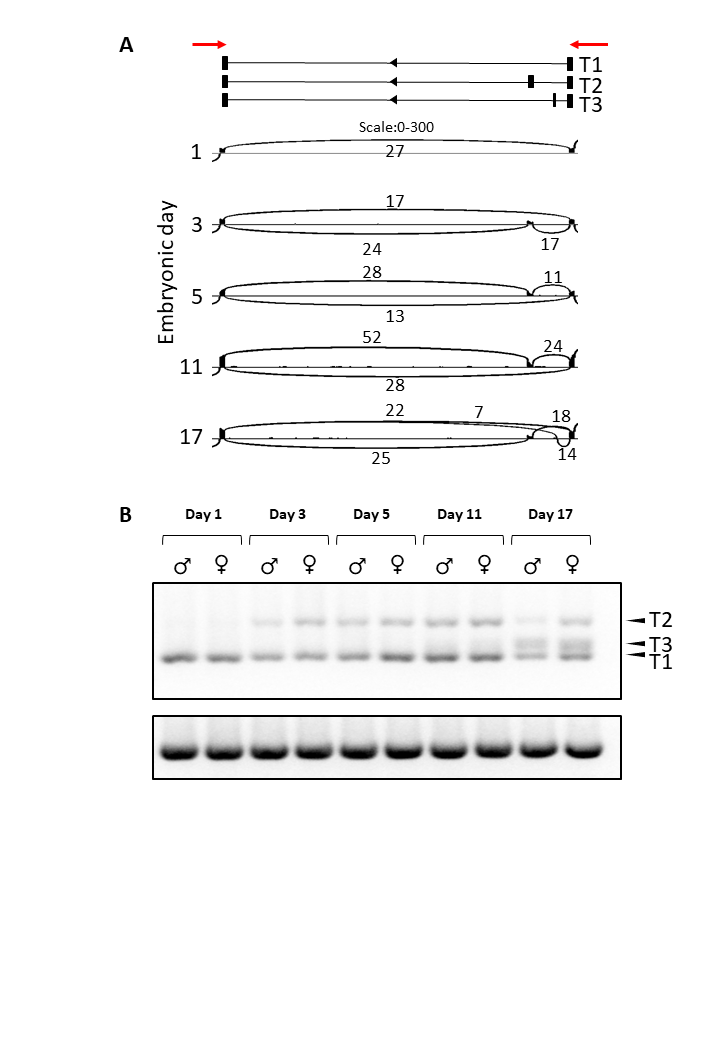

Supplement: Supplementary Figure 5 — Differential splicing during embryonic development. (A) Sashimi plot of skipped exon in gene g35988. In samples from 1-day-old embryos, only one transcript is expressed (T1), in samples of 3- to 11-day-old embryos two transcripts are expressed (T1, T2), and in samples of 17-day-old embryos all three transcripts are expressed (T1, T2, T3). Primers are represented by red arrows. (B) In vitro validation of the different splice variants of clu_6480 in g35988 (marked in black arrows) during embryonic stages (day 1, 3, 5, 11, and 17; top panel). β-actin served as a positive control (bottom panel). [file Image_5.tif]

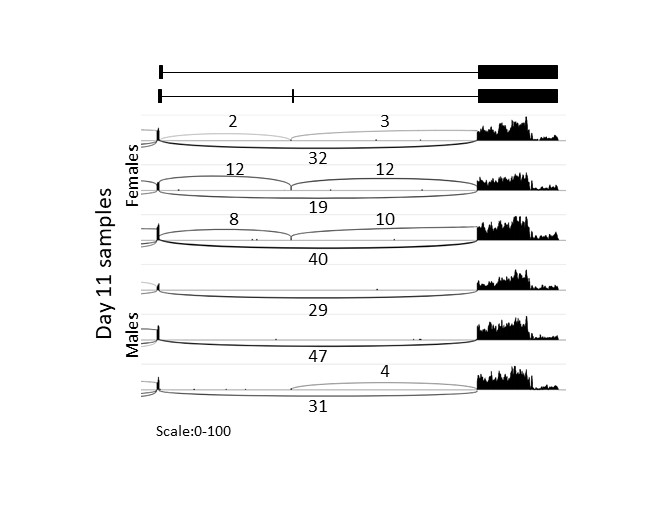

Supplement: Supplementary Figure 6 — Differential splicing between sexes. Sashimi plot of skipped exon in DSE 3387 in samples of 11-day-old embryos. The inclusive transcript (bottom in the diagram) is specific to females. [file Image_6.jpeg]

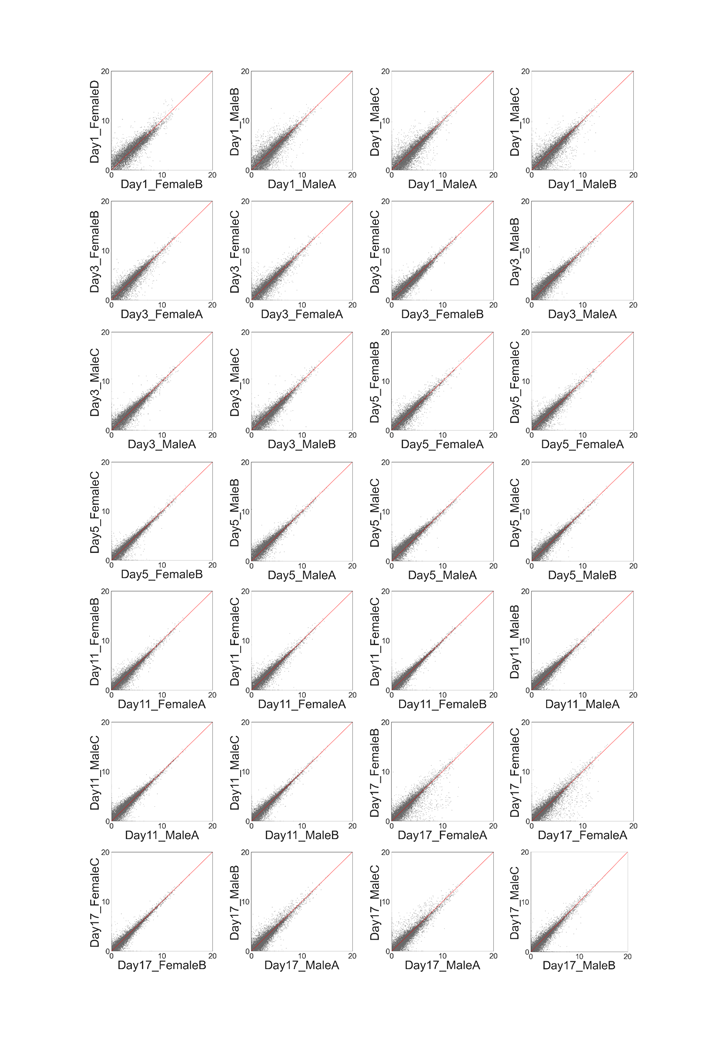

Supplement: Supplementary Figure 7 — Quality control of the samples. Scatter plots of expression levels [log2(TPM)] of all genes between samples of embryos of the same embryonic day and same sex. Each dot is a gene. Red line is the x=y diagonal. [file Image_7.tif]

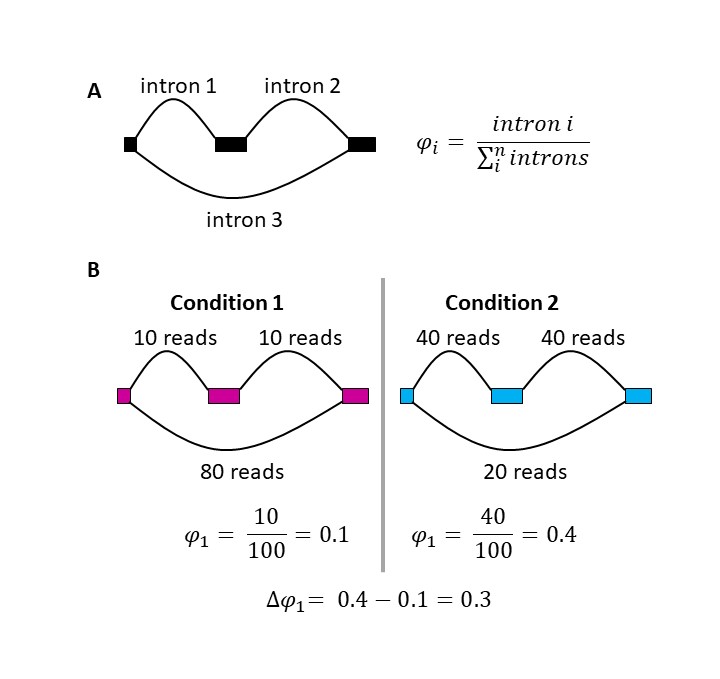

Supplement: Supplementary Figure 8 — Leafcutter nomenclature. (A) A Leafcutter cluster is composed of all introns that share the same start or end location; here, introns 1-3. For each intron in each sample, Leafcutter assigns a percent spliced in (PSI) value, which is the proportion of the junction spanning reads that fall on this intron out of all junction spanning reads that fall on the introns in this cluster. (B) Differential splicing is identified by the difference in PSI between conditions, Δφ. [file Image_8.jpeg]
